# Development of a Modular Board for EEG Signal Acquisition

**DOI:** 10.3390/s18072140

**Published:** 2018-07-03

**Authors:** Tomas Uktveris, Vacius Jusas

**Affiliations:** Department of Software Engineering, Kaunas University of Technology, Studentu St. 50, LT-51368 Kaunas, Lithuania; vacius.jusas@ktu.lt

**Keywords:** biomedical signal processing, electroencephalogram, brain-computer interface, analog front-end, acquisition device

## Abstract

The increased popularity of brain-computer interfaces (BCIs) has created a new demand for miniaturized and low-cost electroencephalogram (EEG) acquisition devices for entertainment, rehabilitation, and scientific needs. The lack of scientific analysis for such system design, modularity, and unified validation tends to suppress progress in this field and limit supply for new low-cost device availability. To eliminate this problem, this paper presents the design and evaluation of a compact, modular, battery powered, conventional EEG signal acquisition board based on an ADS1298 analog front-end chip. The introduction of this novel, vertically stackable board allows the EEG scaling problem to be solved by effectively reconfiguring hardware for small or more demanding applications. The ability to capture 16 to 64 EEG channels at sample rates from 250 Hz to 1000 Hz and to transfer raw EEG signal over a Bluetooth or Wi-Fi interface was implemented. Furthermore, simple but effective assessment techniques were used for system evaluation. While conducted tests confirm the validity of the system against official datasheet specifications and for real-world applications, the proposed quality verification methods can be further employed for analyzing other similar EEG devices in the future. With 6.59 microvolts peak-to-peak input referred noise and a −97 dB common mode rejection ratio in 0–70 Hz band, the proposed design can be qualified as a low-cost precision cEEG research device.

## 1. Introduction

The increasing awareness of brain–computer interfaces (BCI) for brain signal analysis has sparked new interest in electroencephalogram (EEG) acquisition device development. Various rehabilitation [1], entertainment, and even security [2] applications can be implemented by post-processing [3,4,5] such electrical signals recorded from the human scalp. However, developing a BCI is a challenging task due to the noisy and variable nature of the EEG signal itself. The lack of validation, design knowledge, and analysis for such systems impede progress in this field. Even if there were inappropriate trials to use mobile devices for such a problem [6], professional high-quality and high-resolution analog front-ends are required to capture the non-stationary brain signals in microvolt ranges. With the introduction of dedicated EEG low-noise programmable analog-to-digital converters (ADCs) such as the ADS1298, such tasks can be achieved more easily. Professional and high-quality EEG capture systems are available from multiple vendors such as G.Tec and TMSi, etc. Due to their more than four thousand US dollar price (Table 1), these devices are not meant for general public use or entry-level development and, thus, prevent wider BCI adoption and research. Furthermore, there is minimal knowledge of design or operational information on how these devices are actually validated and achieve their proclaimed specifications. Additionally, to our best knowledge, there are no compact EEG systems allowing the scaling and reconfiguration of hardware based on problem requirements (up to 64 or more channels). Achieving this would help manage and reduce complexity and also, minimize runtime costs.

With respect to the previously mentioned problems, this paper presents a new low-cost modular and vertically stackable development board that can be used for entry-level EEG signal acquisition. Furthermore, the proposed design allows the system to be easily scalable and adapted to various EEG tasks, while maintaining significant cost savings. Simple but effective validation methods are presented for acquisition and overall design assessment.

The next sections of this paper give a more detailed review of the proposed solution. Section 2 reviews state-of-the-art designs and approaches found in the literature. Section 3 provides system architecture view and discusses various technical decisions. Methods for system board evaluation and validation, along with experiments are described in Section 4. Result review and comparison with other similar systems are discussed in Section 5. Final conclusions and directions for the future are presented in the last Section 6.

## 2. Related Work

More than a few papers exist that describe the developed prototypes of EEG acquisition systems. F. Pinho et al. [10] presented a computationally powerful, wearable system with 32 active dry electrodes (based on TLC272 precision op-amp) for long-term epileptic patient monitoring. The battery-powered design featured a 24-bit resolution analog-to-digital conversion unit ADS1299 capable of sampling up to 1 ksps. EEG data could be processed real-time on a dedicated 1 GHz ARM CPU or sent to a host PC over Wi-Fi 802.11 b/g for analysis and post-processing. Even though the focus of the work was to create a standalone system with a higher performance CPU, the maximum battery life of 25 h was the main limitation while running under maximum load. Since the device was not optimized for size, this required longer wires and use of active electrodes.

A similar approach was used by S. Feng [11] in designing their EEG acquisition system for solving a steady state, visually evoked potentials (SSVEP) problem. A 16-channel cape for a Beagle Bone Black development board (having an AM3358 ARM Cortex-A8 1 GHz CPU) has been developed with two ADS1299 ADCs and capable of sampling at the speed of 1 ksps. The authors claimed their system was superior due to its provided embedded processing power and ability to work up to 12 h on two lithium batteries. However, while the produced cape consumed only 5% (101.2 mW) of the total required power under maximum load, the use of such system for portable battery powered applications is currently still a big challenge.

B. Senevirathna et al. [12] designed a low-cost 7-channel, small size and battery-powered EEG solution for long-term monitoring of schizophrenic patients. The board used a single ADS1299 ADC that was controlled using SAM G55 microcontroller. Authors claimed their system captured analog data at 250 Hz sample rate and sent it over Bluetooth using 230.4 k baud. The power consumption of 69 mA was reported with all channels active. Similarly, T.T. Vo et al. [13] introduced a low-cost 8-channel EEG recording device for BCI applications. Having an STM32F4 microcontroller, a single ADS1299, and capable of sending data over Bluetooth, the design was dedicated to favor small size and low power usage. A sampling speed of 250 Hz was used to record EEG via wet, gold-cup electrodes. Despite successful validation, both previously mentioned devices lack spatial resolution for EEG and overall board expandability was not considered.

A new re-design for an ECG acquisition system featuring a 24-bit ADS1298 ADC was done by D. Campillo [14]. The author interfaced the 8-channel analog-to-digital converter to an MSP430F5529 microcontroller running at 12 MHz. The presented system board was capable of sampling at 500 Hz rate, the intrinsic channel noise (ICN) was 9 µV, and the common mode rejection ratio (CMRR) was 94 dB. The board was tested for more than 12 h of continuous use. The main limitation of the system for EEG use was the lack of channels for good spatial resolution.

M. Wild et al. [15] presented a tiny 4-channel, in-ear proof of concept EEG acquisition device. Built upon OpenBCI project ideas, authors designed a BCI board with ADS1299 ADC that was interfaced using an Atmega328 microcontroller. The raw EEG data were sent over Bluetooth to remote a PC host for processing. Another 16-channel EEG recording device using dry electrodes has been developed by V. Nathan et al. [16] and tested with SSVEP, P300 speller, and motor imagery BCI tasks. The recorded raw EEG data were sent to the host PC via Bluetooth for final processing.

## 3. System Architecture

This section gives a detailed overview of the main components along with the integration and communication mechanisms that were used to develop the system board.

### 3.1. Analog Front-End

Designing a reliable, high-accuracy, precision analog front-end (AFE) is not a trivial task [17] that is why commercial, off-the-shelf solutions should be considered first. There exist multiple AFE devices in the market that are capable of discretizing the analog EEG signal. Since main brain EEG oscillatory waves propagate in a low-frequency range of 0–40 Hz, a high-sampling performance AFE is not required. Thus, the main focus should be directed to AFEs with a maximum number of supported channels, noise reduction capabilities, and high acquisition resolution. E. Mastinu et al. [18] have compared two popular production grade AFEs—ADS1299 and RHA2216—and found that they give similar results, although slightly better noise performances and higher myoelectric pattern recognition (MPR) accuracy was measured for the ADS1299. D. Acharya et al. reviewed an ADS1299 development board produced by Texas Instruments [19] for EEG task. Based on the evaluation given in their paper the ADS was recommended for EEG acquisition due to low power use, low input referred noise (0.205 μVrms–6.5 μVrms), and overall improvement over provided features in same device segment.

In addition, the OpenBCI development board, which is popular among researchers [20] and entry-level enthusiasts, uses the ADS1299 device for the AFE. According to M. Zieleniewska et al. who compared OpenBCI to a top-class EEG amplifier from TMSi [21], the signal quality was comparable to the commercial EEG amplifier and sufficient for research and advanced BCI applications despite the board and electrode shielding problems.

Due to its extensive features, wide use in the industry, and many applications, the ADS1299 and the alternative ADS1298 were selected as the AFE for the developed system. Free samples of the ADS1298 were acquired from Texas Instruments.

The ADS1298/9 is a device [22] for biopotential measurements and medical instrumentation (electrocardiogram (ECG), electromyogram (EMG) and EEG) with eight low-noise, programmable gain amplifiers (PGAs) and eight high 24-bit resolution Delta-Sigma ADCs. The device has self-test, temperature, and lead-off detection mechanisms. Although similar and designed for the same application, the main differences between the devices are given in Table 2.

### 3.2. Host Microprocessor

There are no general solutions for choosing the host processor for interfacing the AFE. In the literature [10,11,12,13,14,15,16,17,18,19,20,21], depending on the use case and required computational performance, the host processor ranges from microcontrollers to embedded microprocessors with 1 GHz or higher frequency. It is inappropriate to choose high-performing CPU for such battery-powered EEG recording devices. All intensive computations, such as machine learning should be carried out remotely on a host PC. The CPU in this work was chosen so that the required maximum analog front-end sampling speed of 1 ksps and the communication with wireless device modules speed would be reached.

**Figure 1:**
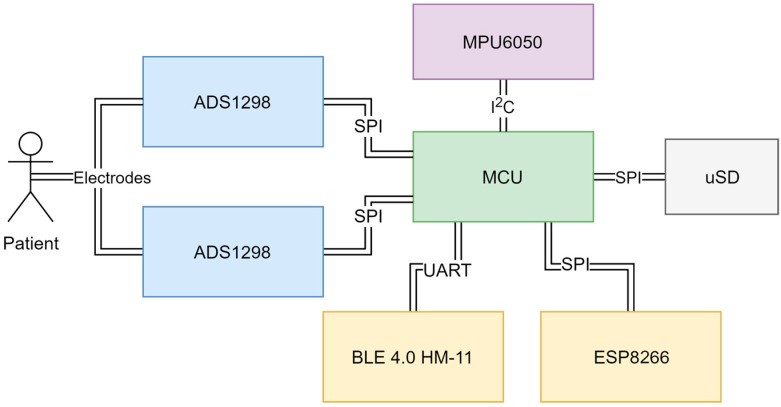
Electroencephalogram (EEG) system board component integration view.

**Figure 2:**
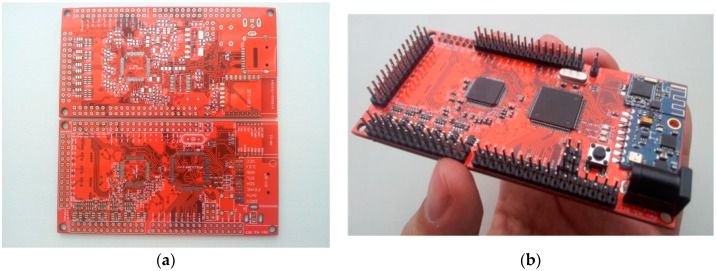
The designed PCB (**a**) Top and bottom of the board; (**b**) Initial version of finished system PCB (dimensions are 10 cm × 5 cm).

For the initial system version (Figure 1), an Atmega2560 microcontroller (MCU) has been selected running at 16 MHz. Interrupt based serial peripheral interface (SPI) communication for ADC sampling and data transmission over wireless connection has been implemented. Two ADS1298 AFEs were tightly packed (top and bottom) on a single 4-layer printed circuit board (PCB) (Figure 2a) giving a total of 16-channel EEG in the standalone system. Additional general purpose inputs-outputs (GPIOs) were broken out by two headers. For wireless communications, two add-on boards were used—Bluetooth 4.0 Low Energy HM-11 (top) and a popular ESP8266 Wi-Fi module (bottom). Additionally, an accelerometer and gyroscope MPU 6050 module controlled over I2C bus was added into the system. Local data storage was implemented using a micro SD card slot. The image of a finished initial system board version can be seen in Figure 2b. The dimensions of the credit card sized board are 10 cm × 5 cm.

**Figure 3:**
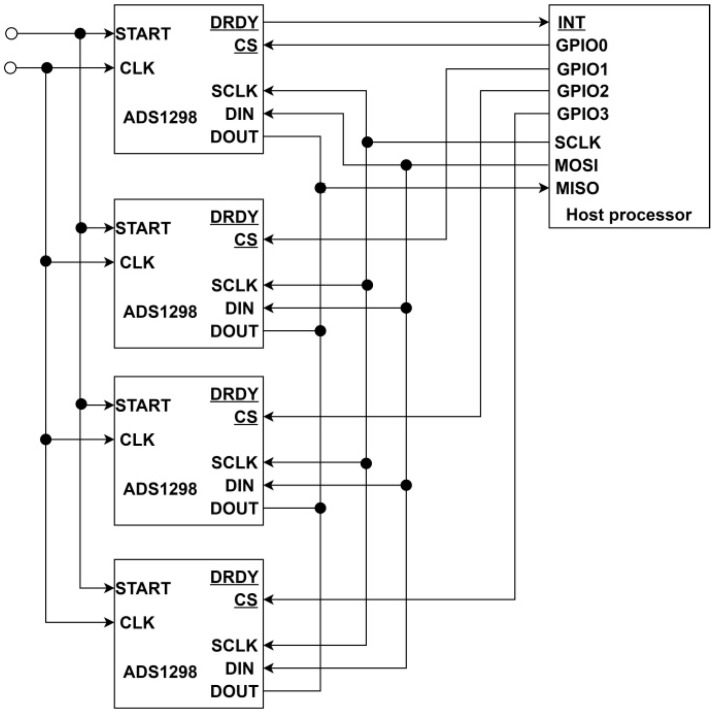
Cascaded view of multiple connected ADS1298 devices.

To achieve expandable and modular architecture, an SPI header was exposed on the PCB for stacking additional boards up to a total count of four, thus, reaching a total of 64 EEG channels. All ADS1298 devices were connected using the cascaded configuration mode (Figure 3). The other supported “Daisy-Chain” configuration type was not acceptable due to limitation–inability to read and write each ADS registers and was not used in this work.

### 3.3. Wireless Communication

To decouple the system board from various AC and other noise sources, the EEG data must be sent to host PC over a wireless connection. Furthermore, wireless transfer is the main solution for replacing long electrode cable braids and limiting the cable swing introduced signal noises and artifacts [23]. Multiple alternatives exist for such a task. The most common approach is to send data over Bluetooth due to the very low power consumption of such technological devices. However, the short connection range and low data rates (baud) are the main bottlenecks of this technique when higher sample rate or higher count of EEG channels are used. Another approach is to use higher bandwidth communication technologies [24] such as Wi-Fi 802.11. By employing Wi-Fi, the bottleneck changes and then the limiting factor is the speed of the MCU.

Both technological approaches were used in the proposed EEG system. The Bluetooth component was implemented using mini HM-11 BLE 4.0 module that is limited to a maximum baud of 230,400. The Wi-Fi component was implemented using ESP8266-12E module via universal asynchronous receiver-transmitter (UART) and SPI interfaces that are limited to maximum baud of 921,600 and MCU speed, respectively. The required baud rate (Table 3) in bits for sending uncompressed EEG data of different sampling speed F_s_ and number of EEG channels N_ch_ can be computed using Equation (1):

(1) $$B_{w}=24\cdot \left(N_{\mathrm{ch}}+1\right)\cdot F_{s}$$

### 3.4. Electrode System and Head Cap

**Figure 4:**
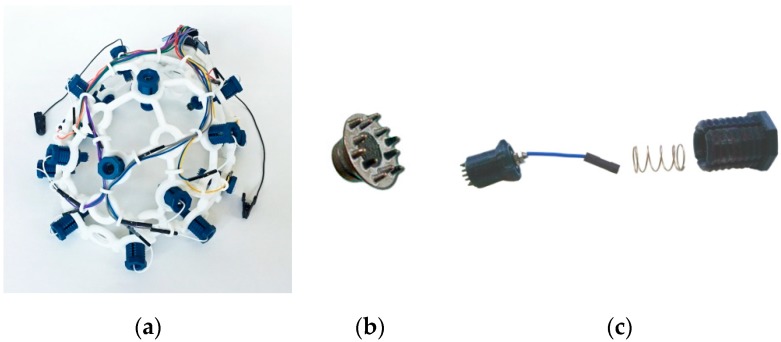
Headcap components (**a**) 3 D printed plastic head cap used for tests; (**b**) Electrode cap from Florida Research Institute; (**c**) plastic electrode holder with a spring system.

A prototype acrylonitrile butadiene styrene (ABS) plastic head cap (Figure 4a) based on the popular open-source Ultracortex OpenBCI model was printed using a 3 D printer and used in tests. The placement of electrodes in the head cap conforms to the international 10–20 electrode system.

Since gel-based electrodes require the application of conductive paste and tend to dry out when used for prolonged times. Reusable dry type EEG electrodes (Figure 4b) from Florida Research Institute were tested instead. Dry electrodes must have good contact with skin to limit resistance to 10 kΩ or less [25]. Pressing the electrode against the skin surface tends to improve the contact with skin. To prevent skin–electrode contact degradation (and thus impedance increase) and due to advances in 3 D printed part usage for EEG [26], a spring tension system (Figure 4c) for each electrode was used in screwable socket type holders to hold the electrode in place.

### 3.5. Accelerometer

The introduction of an accelerometer and gyroscope into the acquisition system allowed the detection of artifacts in the EEG signal (as in Reference [27]) that were introduced due to the movement of the patient. It is not always possible for the subject to stay still for long periods of time. Due to the high component integration, it was optimal to use a pre-existing MPU-6050 module package for the initial version of the system PCB.

### 3.6. Part Costs

The developed system consists of easily obtainable hardware parts. The initial goal was to design the PCB from only the essential pieces that are required for an EEG acquisition board. Table 4 shows the parts used, their prices, and a possible source for building a single board (16-channels) without including manufacturing cost. To build a system with 64 channels, four such boards must be produced. The total price for a single board is about 114 € at the time of writing this paper. This opens more possibilities for researchers and the general public to experiment with BCI. These significant savings come at the expense of performance and have no professional support for hardware and software.

## 4. Evaluation

This section presents the EEG acquisition system board evaluation techniques and tests done to validate the operational correctness. While there are methods to verify the system using high-priced third-party test equipment [28], simpler techniques exist to assess the system. The proposed methods are detailed in further sections. A stacked, four board system was validated with 64 electrodes. Validation tests were done using a high-resolution (HR) mode with PGA gain of one and sample rate of 500 Hz, while a 1 kHz sampling rate was used for Wi-Fi bandwidth evaluation.

### 4.1. Internal ADC Tests

The ADS1298 analog front-end device contains several internal operation modes for validating the internal ADCs. Validation and calibration of ADCs are crucial for correct EEG recordings. The ADS registers (CONFIG1, CONFIG2 and CONFIG3) were programmed to connect internal test signal output to each channel ADC input (INT_TEST = 1). If the channel ADCs are working correctly, the corresponding signal will be seen on each channel output. Three different signal generation modes were tested: Slow 1 Hz square wave (TEST_FREQ = 0), fast 2 Hz square wave (TEST_FREQ = 1), and “DC” mode which allowed a constant high voltage (V_CC_) to be set for each channel.

**Figure 5:**
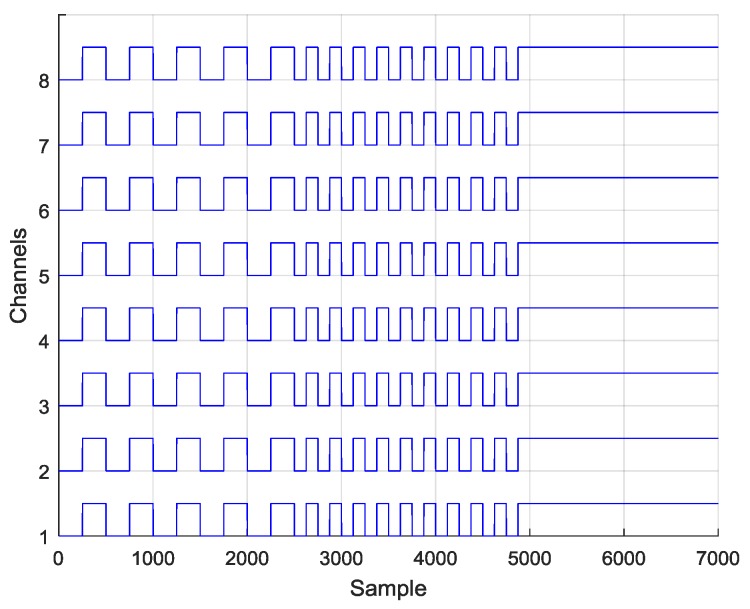
1 Hz, 2 Hz and DC of recorded internal analog-to-digital converter (ADC) test signals.

For each signal type, a several second recording has been captured using a 500 Hz sample rate. An example of the 8-channel data from each test is shown in Figure 5. The recordings presented typical 1 Hz and 2 Hz square waves and DC pattern. This allowed us to conclude that the ADCs were working properly. To validate the system integrity, each time the ADS1298 was started the same signals were used for device calibration.

### 4.2. Lead-Off Detection

Lead-off detection allowed us to validate ADS function to properly recognize the addition or removal of electrodes from the human scalp. This function ensures electrodes have contact with the scalp skin before any EEG recording is made. Lead-off detection has been validated by enabling lead-off detection for each of the EEG channels in ADS registers (LOFF_SENSP = 0xFF). The electrodes were placed on the subject’s scalp, and the status of LOFF_STATP register was checked. The value of 0xFF for the register was expected for proper subject skin contact and a value of 0x00 if all the electrode leads were removed. Additionally, each individual channel was checked using the same routine. The device passed lead-off detection test for all channels successfully.

### 4.3. EEG Capture Software

**Figure 6:**
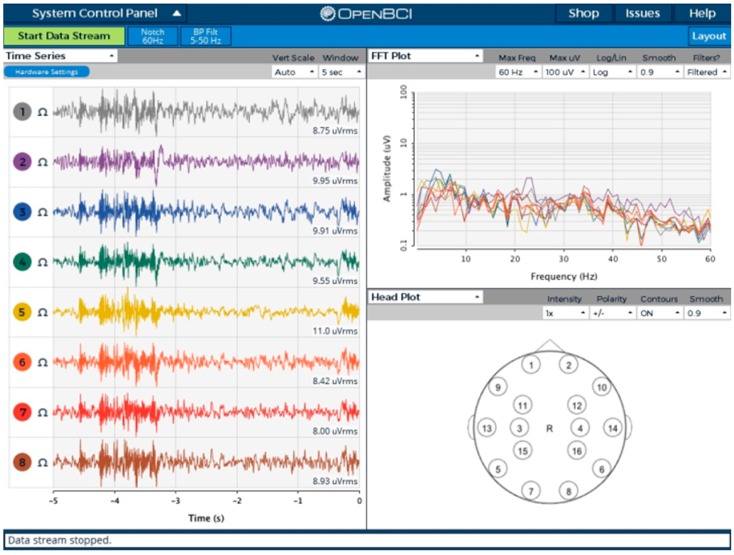
The OpenBCI graphical user interface (GUI) used for validating each ADS1298.

An open-source OpenBCI graphical user interface (GUI) was modified (Figure 6) to support the board developed in this work. The GUI was used for monitoring, recording, and testing purposes. All the raw EEG signal filtering (low-pass, high-pass, and notch filters for 50/60 Hz [29]) was implemented in the software. Support for up to 64 channels has been introduced along with accelerometer data visualization.

### 4.4. Teeth Clenching and Eye Blinks

**Figure 7:**
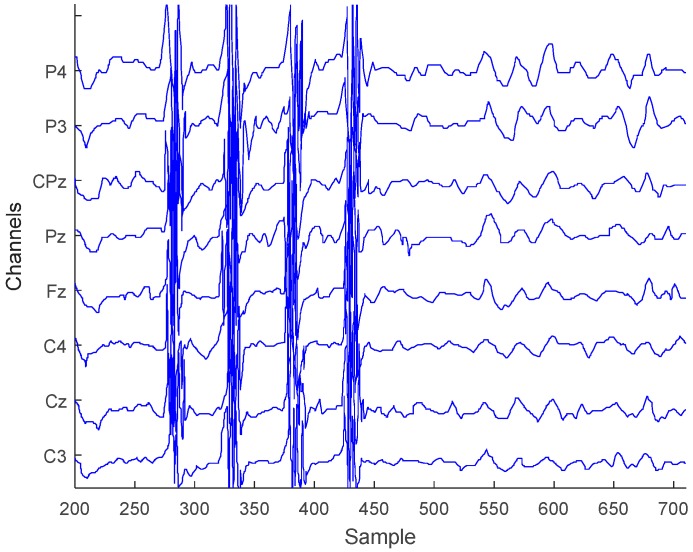
Teeth clenching and eye-blink test EEG signals.

One type of EEG signal artifacts that can be easily captured during a recording session is muscle induced teeth clenching and eye blinks [30]. The existence of these unwanted artifacts allows us to validate the sensitivity of the analog front-end. An EEG recording session has been initiated to see the artifact influence on the system. For this reason, eight electrodes were placed on the subject’s scalp (based on electrode placement system 10–20), and two different states were recorded: Teeth clenching and eye blinks. The resultant EEG trace of the experiment is shown in Figure 7. Clenching artifacts are clearly visible (samples 270–450) while harder to recognize eye blinks have notable periodic behavior (samples 525–700). The recording shows that the analog front-end is susceptible to muscle movement artifacts and also, confirms the sensitivity of the system.

### 4.5. Alpha Waves

Another common technique for EEG recording system validation is to analyze alpha waves [31]. These waves can be recorded in a wakeful human subject during relaxation when the subject’s eyes are closed.

**Figure 8:**
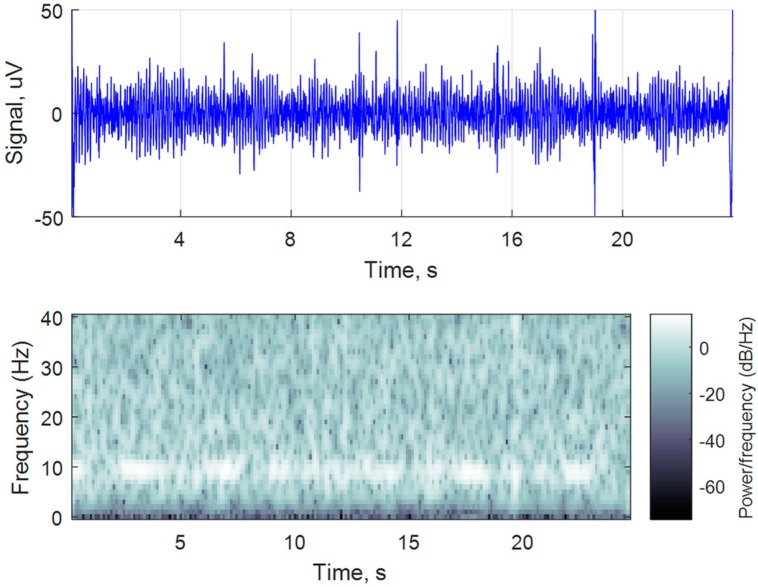
Alpha waves detection from recorded EEG signal.

Detection and recording of alpha waves were tested by connecting electrodes (O1, Oz, O2 from 10–20 electrode placement system) to the subject’s scalp and asking them to relax, open their eyes for 30 s and then, to stay relaxed with closed eyes for one minute. During the closed eyes interval an increased activity in the 7.5–12.5 Hz region in frequency domain showed a typical alpha wave signal (Figure 8) of the brain occipital lobe area. The acquired results proved that the system was able to successfully record EEG signal of such phenomenon.

### 4.6. ECG Signal Detection

**Figure 9:**
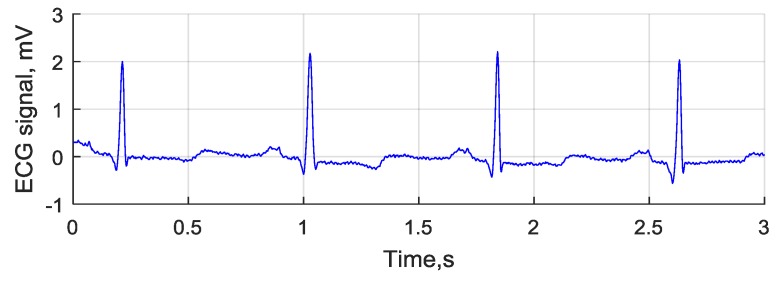
ECG signal recorded using the developed board.

One of the simpler tests that can be initiated to validate any instrumental ADC is to record the activity of the heart (electrocardiogram or ECG). A healthy patient ECG was recorded using three leads. An example of a 78 bpm ECG diagram is shown in Figure 9. Typical periodic QRS complexes are visible in the 2 mV peak signal, which denotes proper functioning of the signal capture front-end.

### 4.7. Input Referred Noise

Input referred noise is each channel’s characteristic showing the noise generated by internal ADS1298 chip circuitry and ADCs. The noise level for each channel was checked by shorting all channels inputs via ADS1298 register CHnSET = 1 (where *n* = 1 to 8) configuration and recording the noise floor for 10 s to a micro SD card using different PGA and sampling rate settings. The averages of each channel noise are given in Table 5.

**Figure 10:**
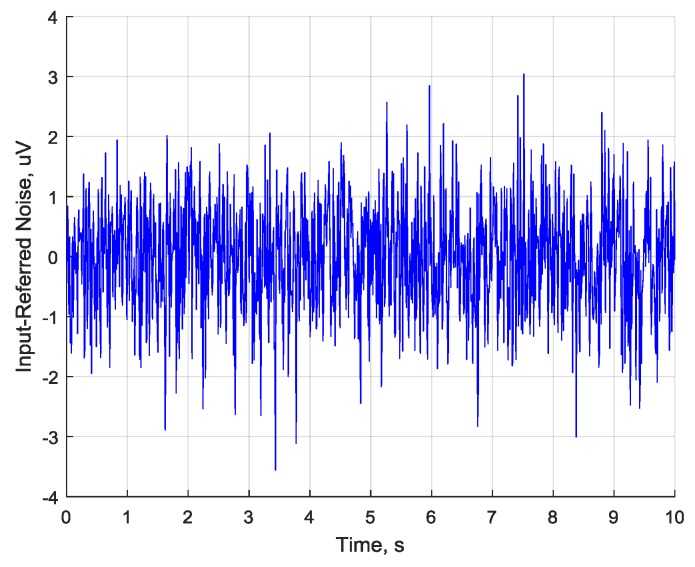
Single channel input-referred noise signal (F_s_ = 1000 Hz, PGA = 3).

An example of noise floor recording for a single channel is given in Figure 10. The average channel input-referred noise value for this signal was 6.59 μV_pp_.

It can be seen that the noise is effectively cancelled as the sampling rate is decreasing (due to averaging done by ADS). A maximum noise decrease of around 1883/5.92 = 318 times has been observed for PGA = 2. High sampling frequency is not required for EEG applications, so rates up to 1 kHz are more than enough to confidently capture brain oscillations in 7–30 Hz range. Further, higher gain value allows a reduction in the input-referred noise. A maximum noise reduction of around 710/48.83 = 14.5 times has been seen in experiments for F_s_ = 16 kHz.

### 4.8. SNR and Precision

**Figure 11:**
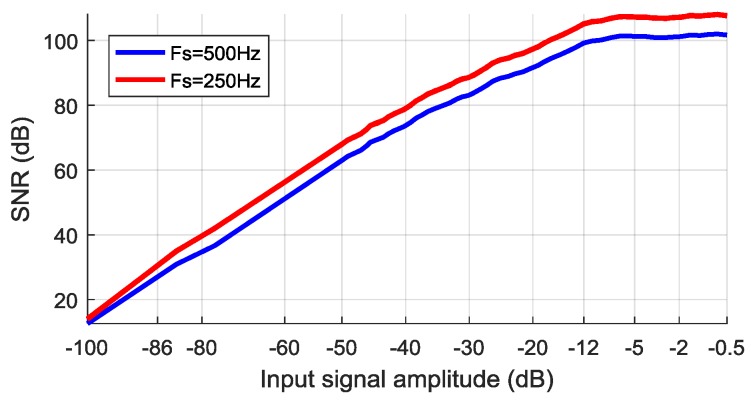
Signal-to-noise ratio (SNR) evaluation results.

Signal-to-noise ratio (SNR) shows the ability of the system to discern effective signals from background noise. When working with EEG signals, it is a requirement to have as high an SNR as possible since the valuable signal is in the same micro-volt range as the noise. The SNR in decibel scale is defined as shown in Equation (2):

(2) $$\mathrm{SNR}=20\;\log_{10}\left(\frac{A_{\mathrm{signal}}}{A_{\mathrm{noise}}}\right),$$

where the A_signal_ and A_noise_ are the root-mean-square (RMS) amplitudes of the signal and noise respectively. To evaluate the SNR of the designed system, an EEG recording experiment was conducted. First, a noise signal of 1 min length was recorded on all ADS1298 channels using 250 Hz sampling speed, and the average noise RMS amplitude was calculated from all data. Next, a known amplitude effective 10 Hz sine signal was generated as input on each channel, and the same length recordings were taken. These acquired signals were used to calculate the average RMS amplitude and finally, the SNR value. The input sine signal amplitude was scaled from 0 dB (100% VCC) to −100 dB (0.001% VCC) to fully capture system behavior for very large and very small signals. The same technique was repeated for 500 Hz sample rate. Results of the experiment are shown in Figure 11. It can be noted, that over 100 dB SNR is reached for input signals whose amplitude is greater than −12 dB (>25% VCC). For typical 10–100 µV (−100 dB to −80 dB) EEG signals the SNR value varies from 12 dB to 35 dB. A lower sampling rate gives higher SNR due to higher ADS1298 signal averaging/oversampling (noise cancellation).

**Figure 12:**
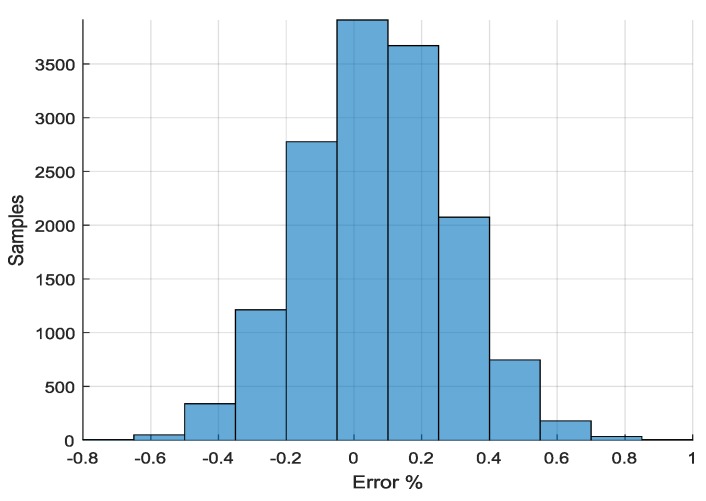
Signal precision for 60 sec recording (F_s_ = 250 Hz, PGA = 6).

ADC precision evaluation used the same previously recorded data. Each of the recorded signal samples was compared with the original sine input signal values to find the conversion error. Results of this experiment can be seen in Figure 12. The average obtained error was 0.07% with a 0.22% standard deviation. The obtained results show a good match with the ones published in official TI ADS1298 datasheet and allows us to qualify the system as a properly working device.

### 4.9. Common Mode Rejection Ratio

The ability to reject common mode signal is crucial for EEG recording systems. A higher common mode rejection ratio (CMRR) ensures that less common-mode signals will appear in the measurements. CMRR is a property of a differential amplifier [32]. The output of such an amplifier can be modeled as a sum of differential and common mode components as shown in Equation (3):

(3) $$V_{\mathrm{out}}=A_{d}V_{\mathrm{in}}+A_{\mathrm{cm}}V_{\mathrm{cm}},$$

where the A_d_ is the differential and A_cm_ is common mode gains expressed as Equations (4) and (5), respectively:

(4) $$A_{d}\approx V_{\mathrm{out}}/V_{\mathrm{in}},$$

(5) $$A_{\mathrm{cm}}\approx V_{\mathrm{out}}/V_{\mathrm{cm}}$$

where

(6) $$V_{\mathrm{in}}=\left(V_{p}-V_{n}\right),$$

(7) $$V_{\mathrm{cm}}=\left(V_{p}+V_{n}\right)/2$$

with V_p_ being the voltage on positive input and V_n_ being the voltage on negative input. While $A_{\mathrm{cm}}=0$ and $A_{d}\to \infty$ for ideal amplifiers. In real applications the $A_{\mathrm{cm}}\neq 0$ and $A_{\mathrm{cm}}\ll A_{d}$. Equation (5) equality holds only when the same common mode signal is fed to both amplifier inputs as the differential gain component is eliminated due to $V_{\mathrm{in}}\to 0$. CMRR can be calculated by evaluating Equation (8):

(8) $$\mathrm{CMRR}=20\log\left(\frac{A_{d}}{A_{\mathrm{cm}}}\right).$$

**Figure 13:**
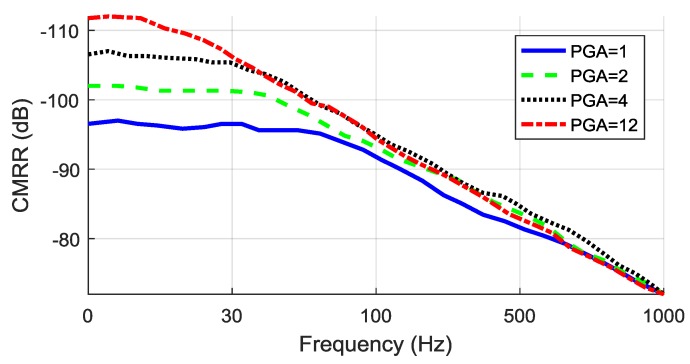
Common-mode rejection ratio (CMRR) measurement results.

The test was performed by connecting each channel differential inputs INxP, INxN (for $x=\bar{1,8}$) and generating an external fixed frequency sine input common mode signal. The voltages were measured, and the average CMRR computed using Equation (8). The test was done for different gain values 1, 2, 4, 12 and frequencies FROM 1 Hz to 1 kHz. Results of CMRR evaluation can be seen in Figure 13.

Test results show that the increase in gain from 1 to 12 allows a ~15 dB better rejection ratio for 1–10 Hz signals to be achieved despite a much quicker decline seen from 15 Hz to 1 kHz. It should be noted, that the smallest gain value of one provides a stable CMRR of ~97 dB for signals up to ~70 Hz. To achieve the highest stable CMRR for EEG signals in 7–30 Hz frequency range, a gain value of four should be used.

## 5. Discussion

The implemented system exposes similar characteristics to other state-of-the-art implementations while introducing new expandability features. The summary of functionality and comparison between other systems found in the literature is given in Table 6.

It should be noted, that most of the systems are designed to be non-expandable/modular from the start. Having a configurable system allows scale on demand and control to be achieved, minimizing the complexity for each problem. Thus, modularity has been taken into account while designing the proposed system. Since ADS1298 can to be easily cascaded, such chip property was exploited.

Furthermore, EEG applications require a moderate number of electrodes to reliably capture brain oscillations. High-end commercial systems are capable of recording 256 channels EEG. However, this creates a significant complexity and is harder to analyze and process later. The proposed design incorporates a configurable number of available channels, up to 64 (four stacked boards) while starting from eight channels (single sided board). Such an electrode count is commonly used in literature for capturing EEG data with adequate head scalp coverage.

Some of the compared systems use active electrodes instead of passive or active shielding to remove cable swing/movement induced artifacts. The 3D printed head cap allows the minimization of the length of cables and to fix them into position, thus, also limits movement-related artifacts. To fully suppress such artifacts, a switch from passive to active electrodes should be made. Since ADS1298 is not designed to work with an external pre-amplification stage, other AFE solutions will be necessary.

The maximum sampling resolution is denoted by the AFE used for each device. Currently, a lot of the devices use a 24-bit AFE to record fine details of the EEG signal. However, it is hard to reach such high discretization resolution due to various PCB designs and physical issues. So, the real resolution is usually much lower due to noise in the least significant bits (LSBs).

Depending on the application the EEG signal processing can be done online or offline. Since mobile EEG devices usually run off battery power (to avoid additional common-mode and other noise from power sources), the power usage must be minimized. Selection of a low-power microprocessor, such as Atmega2560, running at 16 MHz and drawing ~30 mA@5 V on full load still allowed the required 1 kSPS bandwidth to be handled from 8x ADS1298. The power consumption of 8x ADS1298 was ~48 mA@5 V and MPU 6050 accelerometer ~2 mA@5 V, while the most energy was wasted for the Wi-Fi connection ~170 mA@3.3 V. This added up to 250 mAh for the whole four board stack running at maximum load. With a typical 3000 mAh LiPo battery, the system can function up to 12 h.

Maintaining a cost-efficient solution, while providing sufficient quality, is an important topic worth discussion. Since the first-ever EEG devices were made, the most critical analog system part has shrunk and has been embedded inside the silicon chip of the ADC, such as ADS1298. By doing this, higher quality for noise suppression and other parameter controls were achieved. In addition, the integration part got simpler since the microprocessor only needs to interface with the ADC chip. The bill of materials (BOM) has shrunk, and the most expensive part of the EEG system is the ADC chip itself. With the increasing integration level, the future of EEG systems could evolve into a single programmable chip. With this extreme level of integration, further finer control of acquisition system properties could be achieved.

## 6. Conclusions

This paper presented a modular biopotential acquisition system design capable of recording up to 64 EEG channels by exploiting a novel, stackable configuration. Full board tests have been completed, and results showed correct working behavior of each of the system components. The selected system architecture and ADC chip for EEG acquisition proved to be a successful choice for building a compact and modern system. The proposed simple evaluation techniques allowed the system ability to correctly and effectively capture EEG signal to be validated while it also gave needed feedback for further development of the board.

Past internal ADC tests were the initial step for verifying device correctness. An in-range to official ADS1298 datasheet input referred noise value of 6.59 μV_pp_ and average CMRR of −97 dB in 0–70 Hz band was received in other performed tests. The ability to correctly capture EEG alpha waves phenomenon or ECG, also signaled that the system was working as expected. A 12–35 dB SNR for 10–100 uV EEG signals and greater than 100 dB SNR for signals with amplitudes bigger than 25% VCC were measured during experiments. SNR and precision were found to closely match the proclaimed device characteristics as stated in official Texas Instruments datasheet. With a maximum power consumption of ~250 mAh on full load and more than 10 times lower cost (compared to commercial devices), the proposed system can be a portable device for cEEG or ECG acquisition and monitoring.

System comparison with other developed boards found in literature showed similar or better performance. However, compared to commercial grade hardware, the system lacked better noise suppression, and further improvements are needed. Since enhanced noise suppression is required for such high-resolution, AFEs further research and development will be directed towards active electrode implementation and shielding.

## Figures and Tables

**Table 1:** State-of-the-art professional brain–computer interface (BCI) systems.

System	Sampling Speed, Hz	# of Channels	Accuracy	CPU	Electrodes	I/O	CMRR	Price €
g.tec [7] Nautilus	500	64	24-bit, <60 nV (LSB), <0.6 μV RMS	TI DSP	Active-dry/gel	Wireless 2.4 GHz/USB	>90 dB	>4.5 k
g.tec HIamp	38.4 k	256	24-bit, <60 nV (LSB), <0.5 μV RMS	TI DSP	Active-dry/gel	USB	>90 dB	>31 k
TMSi [8] Mobita	2000	32	24-bit, <24 nV	N/A	Passive dry	Wi-Fi IEEE 802.11 b/g	>100 dB	N/A
TMSi [9] Porti	2048	32	22-bit, <1 μV RMS	N/A	Active-shielding	Bluetooth/optic fiber	>90 dB	N/A
TMSi Refa	2048	136	22-bit, <1 μV RMS	N/A	Active-shielding	Optic fiber	>90 dB	N/A

**Table 2:** Main differences of ADS1298/9 devices.

Parameter	ADS1298	ADS1299
Sample Rate (max), ksps	32	16
Input Type	Differential or Single-ended	Differential
Power Consumption, mW	6	41
Min analog voltage, V	2.7	4.75
SNR, dB	112	121
Max programmable gain	12	24
CMRR, dB	−115	−110

**Table 3:** Bandwidth requirements for raw electroencephalogram (EEG) data.

# of EEG Boards	Sampling Speed, Hz	# of Channels	BAUD, BPS	BLE 4.0 UART	ESP8266 UART/SPI
1	250	16	96,000	Yes	Yes/Yes
2	250	32	192,000	Yes	Yes/Yes
2	500	32	384,000	No	Yes/Yes
2	1000	32	768,000	No	Yes/Yes
4	250	64	384,000	No	Yes/Yes
4	500	64	768,000	No	Yes/Yes
4	1000	64	1,536,000	No	No/Yes

**Table 4:** Bill of materials of a single EEG board.

Part#	Item	Usage	Source	Count	Price/Pcs, Eur	Total, Eur
1	4 layer PCB board	Base for mounting SMT devices	Seeed	1	8.00	8.00
2	ADS1298IPAG	Analog front-end chip	Mouser	2	31.00	62.00
3	ESP8266-12E	Wi-Fi module	ebay	1	1.40	1.40
4	MPU6050 GY-521	Accelerometer+gyro module	ebay	1	0.92	0.92
5	Atmega2560	Main CPU	ebay	1	4.20	4.20
6	HM-11	Bluetooth 4.0 module	ebay	1	1.34	1.34
7	SN74LVCC3245	TTL to 3V3 level shifter	Mouser	1	0.98	0.98
8	LM2664	Voltage inverter	Mouser	1	0.73	0.73
9	MCP1825S	5 V LDO/0.5 A	Mouser	1	0.50	0.50
10	MCP1825S-3V3	3.3 V LDO/0.5 A	Mouser	1	0.50	0.50
11	MIC5219-2.5	2.5 V LDO/0.5 A	Mouser	1	0.88	0.88
12	TPS72325	−2.5 V LDO/0.2 A	Mouser	1	2.23	2.23
13	Other components	Capacitors, resistors, diodes, buttons, pin headers, sockets	Mouser	1	30.00	30.00
				**Total (€):**	**82.68**	**113.68**

**Table 5:** Average channel input-referred noise µV_pp_.

Sampling Frequency F_s_, Hz				PGA			
	1	2	3	4	6	8	12
32,000	2876	1883	937	753	617	357	283
16,000	710	285	152	152	101	66	48.83
8000	118	43.90	33.62	33.09	21.65	15.66	11.54
4000	47.49	27.70	15.41	11.49	11.08	10.72	8.94
2000	31.70	13.88	10.25	10.25	6.32	5.70	5.74
1000	16.56	8.67	7.82	6.04	4.55	5.23	3.38
500	14.85	5.92	4.92	4.77	3.94	3.13	2.87

**Table 6:** Comparison with existing boards.

Property	Proposed System	Pinho et al. [10]	Campillo et al. [14]	Boquete et al. [33]	Myung et al. [24]
Modular	Yes	No	No	No	No
Channels	64	32	8	8	16
Sampling frequency, Hz	1000	1000	500	400	512
Electrodes	Passive dry	Active dry	Passive dry	Ag/AgCl adhesive	Wet gel
Resolution, bits	24	24	12	12	24
I/O	BLE 4.0, Wi-Fi 802.11 b/g/n	Wi-Fi 802.11 b/g/n	UART	Zig-bee 802.15.4	Wi-Fi 802.11 d
CPU	Atmega2560	DM3730	MSP430	Atmega2560	STM32F103
Clock frequency, MHz	16	1000	12	16	72
CMRR, dB	−110	−115	−94	-	-
Max gain	12	24	12	10 k	-
Datastore	MicroSD	MicroSD	No	No	No
Local processing	No	Yes	No	No	No
Power, mAh	250	500	-	100	80
